# Impact of the Veneering Technique and Framework Material on the Failure Loads of All-Ceramic Computer-Aided Design/Computer-Aided Manufacturing Fixed Partial Dentures

**DOI:** 10.3390/ma15030756

**Published:** 2022-01-19

**Authors:** Sebastian Hinz, Tobias Bensel, Wolfgang Bömicke, Anders Henningsen, Judith Rudolph, Arne F. Boeckler

**Affiliations:** 1 Department of Prosthodontics, Martin-Luther-University Halle-Wittenberg, Magdeburgerstraße 16, 06112 Halle, Germany; judith23@gmx.de (J.R.); arne.boeckler@web.de (A.F.B.); 2 Department of Prosthetic Dentistry, University Hospital Heidelberg, University of Heidelberg, Im Neuenheimer Feld 400, 69120 Heidelberg, Germany; wolfgang.boemicke@med.uni-heidelberg.de; 3 Department of Oral and Maxillofacial Surgery, University Hospital Hamburg-Eppendorf, 20246 Hamburg, Germany; a.henningsen@uke.de

**Keywords:** zirconium dioxide ceramic, lithium disilicate ceramic, posterior fixed partial dentures, failure loads

## Abstract

Objectives: Zirconia (Y-TZP) ceramics are considered as posterior fixed partial denture (FPD) materials; however, their applications are limited due to chipping. The use of monolithic lithium disilicate (LiDi) glass ceramics in posterior FPDs can be advantageous. This in vitro study aims to compare the loads until failure of posterior Y-TZP-FPDs and LiDi-FPDs before and after aging.

## 1. Introduction

Ceramic materials have attracted increasing attention in dentistry and have emerged as an effective alternative for the fabrication of dental restorations in several applications [1]. Accordingly, the range of applications of all-ceramic systems has increased significantly over previous decades [2,3,4].

Silicate ceramics are suitable for single-tooth restorations in cases with high esthetic demands owing to their excellent optical properties; however, these ceramics do not have the high fracture strengths of zirconium dioxide ceramics, which are about 1200 MPa [5].

Therefore, the use of silicate ceramics is limited to veneers, onlays, and single crowns in the anterior region [6]. Lithium silicate ceramics (LiDi) represent a progression of silicate ceramics. The Li_2_O:SiO_2_ ratio determines the crystalline structure of these materials, thereby indicating which LiDi or lithium aluminosilicate is formed from the raw materials [7]. In LiDi ceramics, the homogeneously dispersed crystalline phase results in an increase in the mechanical strength of the material; therefore, the application of such materials could be extended for the fabrication of full-coverage restorations in the posterior region [8,9,10]. However, the mechanical properties of these materials limit their wide application for replacing teeth [11].

For the frameworks of fixed partial dentures (FPDs), zirconia (Y-TZP) is considered as the preferred material, owing to its superior flexural strength [8,12,13,14]. Several studies have confirmed that the use of zirconia frameworks for short-span FPDs is reliable [15]. These frameworks must be veneered using low-fusion porcelain to achieve functional and esthetic rehabilitation. The medium-term longevity of these frameworks is comparable to the established metal ceramic restorations [16,17]. However, cohesive fractures of the veneering porcelain are frequently observed [18,19,20,21]. This so-called “chipping” occurs due to the different physical parameters of veneering glass, unlike Y-TZP framework parameters, which are independent of the veneering technique [22]. The mechanical strengths and coefficients of thermal expansion must be matched between these materials to avoid chipping of the veneered glass ceramics [23,24].

LiDi glass ceramics do not need to be veneered to imitate the naturalness of human teeth [5]. Single-tooth restorations fabricated from this material can be successfully used clinically in the short-to-medium term [25,26].

For FPDs comprising pressed LiDi, three-unit restorations up to the second premolar as a distal abutment are recommended [27]. Fabricated using the CAD variant, anatomical LiDi-single crowns have exhibited a fracture resistance that is suitable for use as posterior restorations [25] and have demonstrated a higher fatigue resistance than veneered zirconia, which was found to be prone to veneer chipping during cyclic loading [28]. At relatively low temperatures the transformation of stabilized tetragonal zirconia to the monoclinic phase at the surface of the specimen in the presence of water is included by low temperature degradation (LTD) [29]. During artificial aging, the crystalline Li_2_SiO_3_ phase of LiDi-ceramics appears to dissolve more slowly than the glass matrix. This results in in the development of a rough surface [30]. Additionally, artificial aging significantly reduced the strength of the CAD/CAM lithium disilicate [31]. However, in vitro data considering the fracture resistance of CAD/CAM LiDi-FPDs are currently lacking. Previous studies have compared the fracture resistance of posterior monolithic LiDi-FPDs to that of metal ceramic FPDs [32], LiDi pressed on zirconia and monolithic zirconia FPDs, [33] and FPDs constructed of particle-filled composite resin materials. Available in vivo studies on posterior CAD/CAM LiDi-FPDs are typically anecdotal, [34], do not allow for material-specific conclusions [35], or are limited, due to the small number of cases [36]. Therefore, this in vitro study aims to compare the failure loads of three-unit conventionally veneered Y-TZP, pressed-on Y-TZP, and CAD/CAM LiDi-FPDs for the replacement of the first premolar with and without artificial aging. The null hypothesis was that there would be no influence of the FPD type or artificial aging on the failure load.

## 2. Materials and Methods

Herein, for replacing the first premolars, 16 three-unit LiDi-FPDs (IPS e.max computer-aided design (CAD)) and 32 three-unit Y-TZP-frameworks (16 conventionally veneered, IPS e.max Ceram; 16 pressed-on, IPS e.max ZirPress) were fabricated ((computer-aided design/computer-aided manufacturing (CAD/CAM)). Eight FPDs per design group were artificially aged before all FPDs were loaded until failure. The data were analyzed using one- and two-way non-parametric analysis of variance and Tukey’s post hoc tests (α = 0.05).

- Ethics Statement:
  This article does not contain any studies with human participants or animals performed by any of the authors.
- Informed Consent:
  For this type of study, formal consent is not required. The authors have no conflict of interest and no funding to declare.

### 2.1. Fixed Dental Prostheses

**Figure 1:**
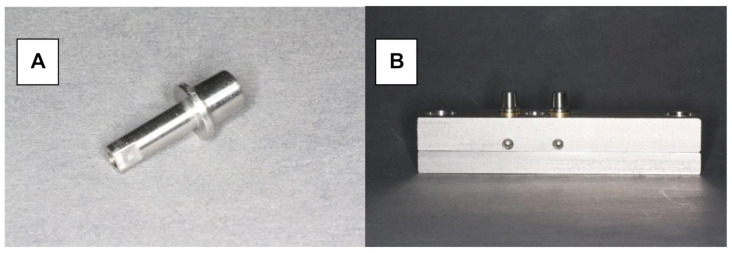
Testing model. (**A**) Stainless steel tooth abutment (height of 6 mm, 3° taper, chamfered preparation with a width of 1.0 mm). (**B**) Testing model with resiliently fixed abutments.

Herein, 48 stainless steel test models were fabricated with identical tooth abutments (6-mm height, 3° taper, and chamfered preparation with a width of 1.0 mm), representing a canine and second premolar (Figure 1A,B). The distance between the abutments in the experimental model was 10 mm. The periodontal ligament was imitated by attaching the abutments to the model base using elastic rubber sleeves (Erkosin, Erkodent, Pfalzgrafenweiler, Germany).

The following FPDs were fabricated and fitted to the test models forming three FPD-type groups (*n* = 16 FPDs per group): monolithic LiDi, conventionally veneered Y-TZP, and pressed-on Y-TZP. All of the FPDs were fabricated using a master FPD constructed of wax as a template.

**Figure 2:**
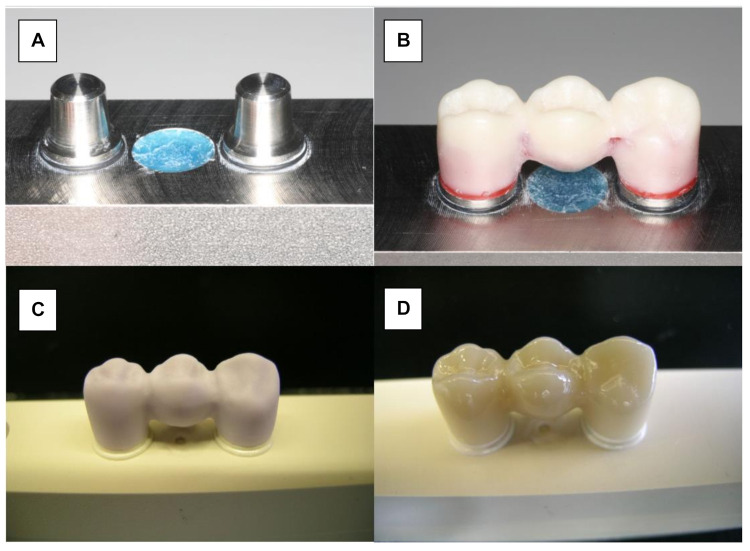
Manufacturing of LiDi-FPDs. (**A**) Testing model. (**B**) Wax-sculptured master FPD. (**C**) Milled framework (IPS e.max CAD blank). (**D**) Characterized and glazed LiDi-FPD.

Monolithic LiDi-FPDs (IPS e.max CAD, Ivoclar Vivadent, Schaan, Liechtenstein) were fabricated using CAD/CAM technology (Figure 2A). The wax master FPD was scanned and served as a design template (Eos Blue Scanner/Cerec 3, Sirona, Bensheim, Germany). Before milling the testing FPDs, the connector dimensions of the wax template were verified to ensure that they conformed to the manufacturers’ recommendations (Figure 2B). According to the manufacturers’ recommendation, the connector must have a cross section of at least 16 mm^2^. The dimensions of the connector between the canine and first premolar were 5.81 mm high and 3 mm wide (19.75 mm^2^). The surface area of the posterior connector was 21.81 mm^2^ (6.38 mm high and 4.37 mm wide). Further processing was performed in accordance with the manufacturers’ specifications. The monolithic restorations were milled (Sirona inLab MC LX, DentsplySirona, Bensheim, Germany) and crystallized at 850 °C (Programat P200, Ivoclar Vivadent; Figure 2C). Finally, glaze firing was conducted at 840 °C (IPS e.max CAD Crystal/Glaze, Ivoclar Vivadent; Figure 2D).

Zirconia frameworks were fabricated using CAD/CAM technology (Organic Zirkon, R+K CAD/CAM Technology, Berlin, Germany). The master FPD was scanned and virtually reduced by 1 mm to obtain the minimum thickness of the veneering ceramics (Scanner D700 and Dental Designer, 3 Shape, Copenhagen, Denmark). In accordance with the manufacturers’ instructions, the frameworks retained a minimum thickness of 0.7 mm and the connector dimensions were 19.75 and 21.81 mm^2^. The frameworks were milled (Organic Zirkon opak/light; Organical 4XT; R+K CAD/CAM Technology) and sintered for 2 h at 1450 °C.

**Figure 3:**
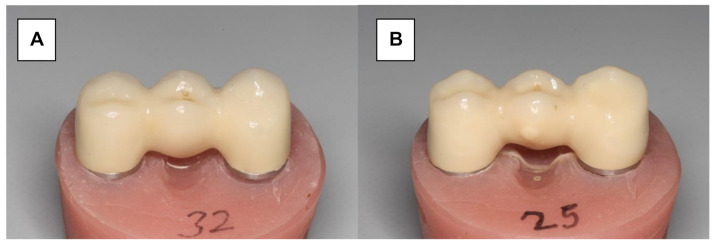
Zirconia three-unit FPD. (**A**) Conventionally veneered with low-fusing veneering porcelain. (**B**) Veneered using the press-on technique.

Herein, 16 Y-TZP frameworks were conventionally veneered and glazed using a low-fusing nano-fluorapatite glass ceramic (IPS e.max Ceram/IPS e.max Ceram Glaze Powder, Ivoclar Vivadent) (Figure 3A). The veneering process was performed in several layers and ceramic firings (Programat 500, Ivoclar Vivadent). A silicone mold (Sheraduett Soft, Shera GmbH, Lemfoerde, Germany) derived from the master FPD was used to ensure the identical shapes of the resulting testing FPDs.

Additional 16 Y-TZP frameworks were veneered using the press-on technique (Figure 3B). The frameworks were scanned (CAD System 3Shape Scanner D700, 3Shape A/S, Copenhagen, Denmark) and superimposed with the scanned master FPD using “Dental Designer” (3Shape A/S, Copenhagen, Denmark). The veneering parts were milled in wax (Organic Wax, R+K CAD/CAM Technology) and attached to the frameworks. The basal shape of the pontic was completed manually using the silicone mold. The FPDs were embedded in a special investment material (IPS PressVEST, Ivoclar Vivadent) and the wax was expelled thermally. Frameworks were over-pressed with fluorapatite glass ceramics (IPS e.max ZirPress Ivoclar Vivadent, color: LT A3) at 770 °C (ceramic oven: Programat EP 500, Ivoclar Vivadent), and the FPDs were finalized using glaze firing (IPS e.max Ceram Glaze Powder and Programat EP 500, Ivoclar Vivadent, Ellwangen, Germany).

All the FPDs were cemented with glass ionomer cement (Ketac Cem, 3M Espe, St. Paul, MN, USA.) in a standardized manner (5 N, 15 min) using a universal testing machine (ZWICKI TMZW, Zwick GmbH & Co KG, Ulm, Germany). Before cementing, the intaglio surface was airborne-particle abraded with 115-µm alumina at 0.15 MPa.

### 2.2. Artificial Aging

**Figure 4:**
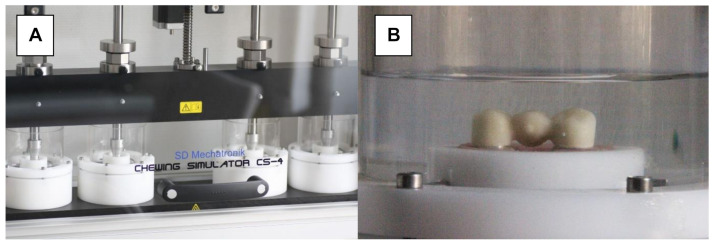
Artificial aging. (**A**) Chewing simulator. (**B**) FPD submerged in artificial saliva during cyclic mechanical loading.

Half of the FPDs in each design group were exposed to 1.2 million biaxial chewing cycles (chewing simulator CS-4, SD Mechatronic, Holzkirchen, Germany; Figure 4A) at a temperature of 37 ± 2 °C with mean forces of 50 N and a frequency of 1.6 Hz in artificial saliva (Glandosane Neutral Spray, Cell Pharm GmbH, Bad Vilbel, Germany, ratio 1:2). A steatite ball (diameter: 5 mm) loading the pontic centrally at an angle of 30° transferred the resulting forces (vertical speed: 50 mm/s; lateral speed: 60 mm/s; vertical stroke: 3 mm; and lateral stroke: 0.7 mm). Subsequently, the FPDs were cycled thermally for 10,000 cycles, alternating between tempered water of temperatures 5–55 °C (WEDC1V, Version 2.5, Willytec) (Figure 4B). This aging scenario simulated a wearing period of approximately 5 years [37]. The artificially aged FPDs were visually inspected for damage before being loaded to failure.

### 2.3. Load-to-Failure

The load-to-failures of the cemented FPDs were determined using a universal testing machine (ZWICKI TMZW, Zwick GmbH & Co. KG, Ulm, Germany). To achieve this, the pontic was vertically loaded with constantly increasing force (preload: 10 N; and testing speed: 1 mm/min). A piece of tin foil (1-mm thick) was placed between the specimen and plunger (diameter: 4 mm) of the universal testing machine to avoid stress peaks. Failure was defined as a 10% drop in force during static loading (test Xpert II, Zwick GmbH & Co. KG, Ulm, Germany), which coincided with the complete fracture of the examined restorations. Specimens showing complete fracture after only artificial aging were considered to exhibit a failure load of 50 N. If fracture gaps or the chipping of the veneering ceramic could have been visually or acoustically recognized, the determination of this incident was confirmed subjectively as an additional notice (data not shown).

### 2.4. Statistics

Statistical analyses were performed using an IBM SPSS 25.0 (IBM, Incorp., Armonk, NY, USA). As normal distribution could not be assumed (Kolmogorov–Smirnov test, *p* ≤ 0.024), the collected data were analyzed using two- and one-way non-parametric analysis of variance (ANOVA) and Tukey’s post hoc tests [38]. Homoscedasticity of the data was accepted (Levene test, *p* = 0.257). The level of significance was set to 5% (α = 0.05).

## 3. Results

The mean failure loads of LiDi-FPDs were 1293 ± 237 and 996 ± 516 N (aging; *p* = 0.712), 1609 ± 427 and 1685 ± 480 N (*p* = 1.000) for e.max ZirPress-FPDs, and 1541 ± 438 and 1557 ± 643 N (*p* = 1.000) for e.max Ceram-FPDs. After aging, higher failure loads were observed for e.max ZirPress-FPDs than those for LiDi-FPDs (*p* = 0.042).

### Load-to-Failure

Inspection of the artificially aged test specimens prior to failure load testing revealed the complete fracture of one LiDi-FPD, and cohesive chipping in the area of the palatal cusp of the pontic was noted in two other LiDi-FPDs. No chipping was observed for the Y-TZP-FPDs, but the initial veneer cracking in the pontic was found for two pressed-on Y-TZP-FPDs and three conventionally veneered Y-TZP-FPDs. The failure load of LiDi-FPD showing complete fracture after artificial aging was set to 50 N for statistical evaluation, whereas all the other FPDs could be tested using static failure loading.

**Figure 5:**
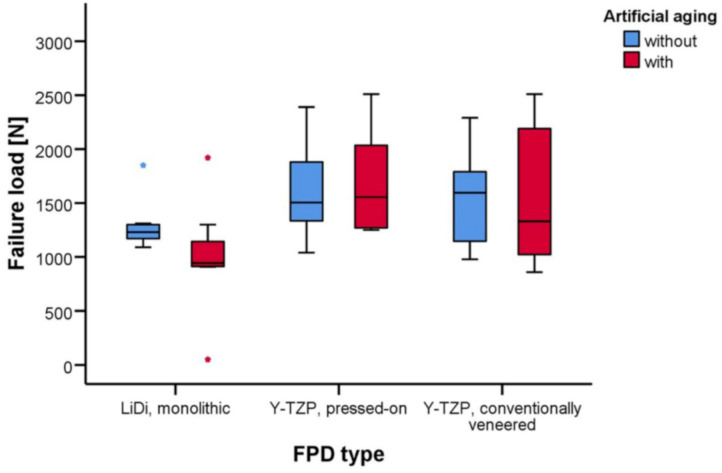
Whisker and box plots of the failure loads [N] for fixed partial dentures constructed of monolithic LiDi, pressed-on Y-TZP, and conventionally veneered Y-TZP with and without artificial aging.

The failure loads of the FPDs are provided in Table 1 and shown in Figure 5. The mean failure loads in the testing ranged between 996 ± 516 N for the artificially aged LiDi-FPDs in the minimum and 1685 ± 480 N for the artificially aged pressed-on Y-TZP-FPDs in the maximum. Two-way non-parametric ANOVA revealed the statistically significant effects of the FDP type (*p* = 0.008), while both artificial aging (*p* = 0.397) and the interaction of these terms (*p* = 0.511) were not statistically significant. In the pairwise tests, the failure loads for FPDs of one type did not differ in a statistically significant manner between test specimens with and without artificial aging (*p* ≥ 0.712). Considering the unaged FPDs, there were no statistically significant differences in failure loads between the different FPD types (*p* ≥ 0.677). However, for the artificially aged FPDs, statistically higher failure loads were found for pressed-on Y-TZP-FPDs than those for monolithic LiDi-FPDs (*p* = 0.042), while other comparisons in the group of the aged FPDs were again not statistically significant (*p* ≥ 0.262) (Table 2).

## 4. Discussion

All-ceramic restorations have excellent esthetic characteristics. Y-TZP-ceramics are high-performance ceramics that have been established for the framework of posterior FPDs. However, chipping is a major clinical problem when using veneered FDPs. CAD/CAM technology has made it such that monolithic LiDi-ceramics do not need to be veneered.

In this study, three-unit Y-TZP-FPDs and LiDi-FPDs replacing the first premolar with and without artificial aging were compared considering their failure load in an in vitro model. The performance of posterior LiDi-FPDs was comparable to that of the veneered CAD/CAM-fabricated FPDs. The average load-to-failure values of the investigated LiDi-FPDs were 1293 N without aging and 996 N with aging, which is comparable to other results [28]. Moreover, for the LiDi-FPDs without aging, the minimum failure load was 1090 N. Therefore, the requirement was met that posterior all-ceramic FPDs should possess an initial fracture load of 1000 N [39,40].

The load-to-failure values for the pressed-on Y-TZP-FPDs ranged from 1609 (without aging) to 1685 N (with aging). The conventionally veneered Y-TZP-FPDs achieved results ranging from 1541 N (without aging) to 1557 N (with aging). This outcome is confirmed by other studies that have tested these types of FDPs in vitro [41,42,43].

Artificial aging is usually part of in vitro studies. The simulated aging scenario of a wearing period of approximately five years was used [44]. The temperature changes generate stresses in the ceramic. In addition, here, a cyclic loading force of 50 N was applied to simulate clinical loading conditions. This approach is similar to that used in other studies [37,45,46]. A value of 50 N was chosen as the minimum because this force is below realistic clinical stress levels in the posterior area [47].

Artificial aging had no significant influence on the tested FPDs. However, previous studies have determined different results. It is possible that a more extensive aging process would have a stronger influence on the samples. This could lead to significant differences. The load-to-failure values of LiDi-FPDs (Empress 2, Ivoclar Vivadent) were observed to decrease from 1832 to 410 N after undergoing chewing simulations [37]. Moreover, the load-to-failures of four-unit zirconia FPDs were previously observed to decrease up to 40% after artificial aging [48]. Possible causes of these phenomena could be the decreasing threshold for critical crack growth in an aqueous solution. However, leakage of water when obtaining the framework remains uncertain. Additionally, changes in temperature have been suggested to induce mechanical stresses, thereby decreasing the load-to-failure [48]. For the current study, it can be assumed that the dimensioning of the restoration was sufficiently strong that the selected aging setup only caused stress that was below the fatigue strength of the material [49]. A higher load (e.g., 150 N instead of 50 N) during artificial aging could have a different effect [37].

The test method chosen in this study—the elastic support of tooth abutments during aging and testing—and the use of a tin foil between the plunger and the occlusal ceramic surface during loading probably ensured a continuous increase in stress and minimized additional stress peaks. Compared to a rigid tooth abutment support and failure loading without an occlusal foil, the initiation of cracks can be reduced. In partial accordance with clinical use, this could explain the approximately constant fracture forces that are independent of the aging process. The influence of patient-specific conditions, such as occlusion and oral hygiene, should not be ignored considering real oral cavity conditions [50,51].

The present study verified that there were no significant differences regarding the load-to-failure between the two tested veneering techniques. According to cohesive fractures of the veneering (cracks and chipping), the over-pressed zirconia FPDs performed better than the conventionally veneered Y-TZP-FPDs (two over-pressed zirconia FPDs vs. three conventionally veneered Y-TZP-FPDs with initial cracking after aging). In this study, chipping did not occur for the examined Y-TZP-FPDs. Three LiDi-FPDs displayed cohesive fractures after chewing simulations in different fracture patterns (two FPDs demonstrated cohesive chipping of the palatal cusps while one demonstrated a complete fracture). These findings are in contrast with those of previous in vivo studies [23,25,48,52]. The premature total failure of the LiDi-FPDs could be due to the incorrect processing. Conversely, the chipping of conventionally veneered Y-TZP-FPDs has been frequently reported in clinical settings [34,35,53]. As there is currently very little data available on the fracture strength of CAD/CAM-manufactured LiDi-FPDs, the results of this study will be relevant to future clinical research, thus aiming to address this present gap in data.

However, despite the fact that artificial aging has been used in this in vitro study, it can hardly imitate the complex structure of the oral cavity and a possible microbiological effect on the framework structure cannot be excluded. In addition, the sample size in this study was reduced to a statistically sensible minimum. Due to the explorative character of the present study, p-values should be interpreted descriptively. It is a weakness of this study that an a priori sample size calculation did not take place. Instead, a convenience sample was chosen for which statistically significant effects had been found in previous studies [42,54,55,56].

In summary, FPDs constructed of LiDi glass ceramics performed well compared to veneered zirconia FPDs. The results of load-to-failure tests confirm the applicability of three-unit LiDi-FPDs for replacing first premolars.

## 5. Conclusions

Within its limitations, this in vitro study indicates that the failure load of LiDi-FPDs may be sufficiently high for the prosthetic replacement of a first premolar. Conducting clinical trials to confirm this finding appears warranted.

## Figures and Tables

**Table 1:** Failure loads [N] of fixed partial dentures with and without artificial aging.

FDP	Artificial Aging	Mean (SD)	Min–Max	Median (25th/75th Percentile)
LiDi, monolithic	No	1293 (237)	1090–1850	1230 (1170/1300)
Y-TZP, pressed-on		1609 (427)	1040–2390	1505 (1335/1880)
Y-TZP, conventionally veneered		1541 (438)	978–2290	1595 (1145/1790)
LiDi, monolithic	Yes	996 (516)	50–1920	943 (912/1143)
Y-TZP, pressed-on		1685 (480)	1250–2510	1555 (1270/2035)
Y-TZP, conventionally veneered		1557 (643)	859–2510	1330 (1022/2190)

LiDi, lithium disilicate.

**Table 2:** Multiple Comparisons.

Groups		Mean (SD)	Sig.	Min–Max
LiDi, not aged	Zirconia, over-pressed, not aged	9.62 (6.492)	0.677	−9.76–29.01
	Zirconia, conventionally veneered, not aged	6.38 (6.492)	0.921	−13.01–25.76
	LiDi, aged	−9.25 (6.492)	0.712	−28.63–10.13
	Zirconia, over-pressed, aged	10.63 (6.492)	0.580	−8.76–30.01
	Zirconia, conventionally veneered, aged	5.00 (6.492)	0.971	−14.38–24.38
Zirconia, over-pressed, not aged	LiDi, not aged	−9.62 (6.492)	0.677	−29.01–9.76
	Zirconia, conventionally veneered, not aged	−3.25 (6.492)	0.996	−22.63–16.13
	LiDi, aged	−18.87 (6.492)	0.060	−38.26–0.51
	Zirconia, over-pressed, aged	1.00 (6.492)	1.000	−18.38–20.38
	Zirconia, conventionally veneered, aged	−4.62 (6.492)	0.979	−24.01–14.76
Zirconia, conventionally veneered, not aged	LiDi, not aged	−6.38 (6.492)	0.921	−25.76–13.01
	Zirconia, over-pressed, not aged	3.25 (6.492)	0.996	−16.13–22.63
	LiDi, aged	−15.63 (6.492)	0.177	−35.01–3.76
	Zirconia, over-pressed, aged	4.25 (6.492)	0.986	−15.13–23.63
	Zirconia, conventionally veneered, aged	−1.38 (6.492)	1.000	−20.76–18.01
LiDi, aged	LiDi, not aged	9.25 (6.492)	0.712	−10.13–28.63
	Zirconia, over-pressed, not aged	18.87 (6.492)	0.060	−0.51–38.26
	Zirconia, conventionally veneered, not aged	15.63 (6.492)	0.177	−3.76–35.01
	Zirconia, over-pressed, aged	19.88 * (6.492)	0.042	0.49–39.26
	Zirconia, conventionally veneered, aged	14.25 (6.492)	0.262	−5.13–33.63
Zirconia, over-pressed, aged	LiDi, not aged	−10.63 (6.492)	0.580	−30.01–8.76
	Zirconia, over-pressed, not aged	−1.00 (6.492)	1.000	−20.38–18.38
	Zirconia, conventionally veneered, not aged	−4.25 (6.492)	0.986	−23.63–15.13
	LiDi, aged	−19.88 * (6.492)	0.042	−39.26–−0.49
	Zirconia, conventionally veneered, aged	−5.63 (6.492)	0.952	−25.01–13.76
Zirconia, conventionally veneered, aged	LiDi, not aged	−5.00 (6.492)	0.971	−24.38–14.38
	Zirconia, over-pressed, not aged	4.62 (6.492)	0.979	−14.76–24.01
	Zirconia, conventionally veneered, not aged	1.38 (6.492)	1.000	−18.01–20.76
	LiDi, aged	−14.25 (6.492)	0.262	−33.63–5.13
	Zirconia, over-pressed, aged	5.63 (6.492)	0.952	−13.76–25.01

* The mean difference is significant at the 0.05 level.

## Data Availability

The data used to support the findings of this study may be released upon an application to the Department of Prosthodontics, Martin-Luther-University Halle-Wittenberg, which can be contacted through Arne Boeckler, Department of Prosthodontics, University Hospital Halle, Magdeburger Straße 16, 06112 Halle, Germany.
